# A summary of relationships between alternative splicing and breast cancer

**DOI:** 10.18632/oncotarget.17727

**Published:** 2017-05-09

**Authors:** Zhang Xiping, Wei Qingshan, Zhao Shuai, Yang Hongjian, Ding Xiaowen

**Affiliations:** ^1^ Department of Breast Surgery, Zhejiang Cancer Hospital, Hangzhou 310022, Zhejiang Province, China; ^2^ Cataloging Department, Library of Xi'an Jiaotong University, Xi'an 710049, Shaanxi Province, China

**Keywords:** alternative splicing, breast cancer, spliceosome, variants

## Abstract

Alternative splicing (AS) is the process of combinatorial rearrangement of parts of exons, and/or parts of introns into mature RNA to result in a multitude of transcripts. AS is a biological process through which organisms produce as many protein variants as possible by a limited genetic resource. It plays an important role in growth and development of the organisms. Over the past few years, alternative splicing has been discovered to be critical for genesis and development of malignant tumors, including breast cancer. If the relationships between AS and breast cancer can be discussed more deeply, it will be helpful for better diagnosis, judging prognosis and intervening with breast cancer. In this paper, the relationships between AS and breast cancer are elaborated from different angles, in hope that this summary is beneficial for readers to understand the roles of AS and breast cancer.

## INTRODUCTION

Alternative splicing (AS) is a mechanism through which cells generate multiple messenger RNAs (mRNAs) with different functions from a single genomic locus. This is conducted by the inclusion or exclusion of specific exons in pre-mRNA processing. It occurs in nearly all the mammalian genes that consist of multiple exons and is catalyzed by the spliceosome occurs after a protein-coding gene is transcribed into mRNA [1]. It splices a pre-mRNA by multiple ways to finally produce several mRNAs, which are respectively translated by ribosome into different protein variants with various biological functions.

Some researchers suggest that AS has important effects in the genesis and development of breast cancer. Its impacts upon breast cancer may be summarized as follows: Breast cancer may be promoted or inhibited by AS of some genes, (2) Some spliceosomal proteins have significant effects on the breast cancer, (3) Breast cancer is affected by some other RNA-binding proteins. It is helpful for human beings to better intervene with and treat breast cancer by grasping and exploring the above information of the relationship between AS and breast cancer.

## PROMOTING ALTERNATIVE SPLICING OF BREAST CANCER

### Alternative splicing of ERα

ERα66 is the protein obtained through the expression of full-length estrogen receptor alpha (ERα) genes, known as ER-α 66. There are two kinds of gene shear isomers, molecular weight of 46kDa and 36kDa, named ER-α46 and ER-α36 respectively. ER-α 66, ER-α 46 and ER-ß are defined as the ligand activated transcription factor of regulation of estrogen responsive gene transcription in the nucleus. However, ERα46 and ERα36 are produced as two variants owing to different effects of AS. ERα36 responds to both estrogen stimuli and antiestrogens. The transduction of ERα36 originates from signaling cascades of cell membrane to stimulate cell proliferation and perhaps contributes to more aggressive phenotypes of breast cancer [2]. ERα36 has been discovered to be exclusive to the expression of ERα66 [3]. The expression of ERα36 is really related to the downregulated expression of ERα66 [2]. ERα66 may downregulate the activity of the promoters that transcribe ERα36 [4]. ERα66 and ERα36 are in equilibrium in normal tissues although they suppress each other. Once such equilibrium is broken, abnormal proliferation and differentiation of cells would be induced. As a result, breast cancer will be caused.

As a type of antiestrogen drugs, Tamoxifen (TAM) is often used for treating ER positive breast cancer. However, breast cancer is resistant to TAM in many cases [5]. It is necessary to deeply study why breast cancer is resistant to TAM. It will be unfavorable for improving therapeutic effects of TAM if there is a high concentration of ERα36 in breast cancer cells, which indicates that ERα36 is one of causes of the resistance to TAM [6]. In addition, it has been pointed out that the presence of TAM increases the concentration of ERα36 in breast cancer cells, and ERα36 is found to be highly expressed in TAM-resistant breast cancer cells [7]. It is clear that ERα36 is a product of AS of ERα genes. ERα36 can promote breast cancer, and has related to invasiveness of breast cancer and the degree of resistance to TAM.

### Alternative splicing of BRCA1 and BRCA2

The two genes most commonly mutated in hereditary breast and ovarian cancer are the tumor suppressor genes BRCA1 (breast cancer gene 1) and BRCA2 (breast cancer gene 2). The probability of BRCA1 or BRCA2 mutation depends on many factors, such as frequency of related disease, age at onset and the affected organs (including breast and ovary) [8]. Mutations of BRCA1 or BRCA 2 genes can increase the risk to develop breast cancer. The incidence of breast cancer is high in some families whose BRCA1 or BRCA 2 is mutant [9]. Many variants of BRCA1 or BRCA 2 have been detected in patients with breast cancer, but their related l significance is still unclear. Some of these invariants are possibly involved in the process of AS. Sanz DJ et al have investigated that the effect of AS of BRCA1 or BRCA 2 on hereditary breast or ovarian cancer (HBOC) in Spanish. They found that there is an important fraction of DNA variants of BRCA1 or BRCA 2 presents aberrant splicing [10]. Some researchers have confirmed that some splice site variants of BRCA1 and BRCA2 can produce deleterious exonic variants which have relationship with the breast cancer families [11].

In normal tissues, BRCA1 is identified to have several subtypes of mRNA splicing, which are discovered to skip over exons 5, 11 (all or a majority), 2-10, 9-11, 14-17 or 14-18 [12]. Such splicing has no direct connections with the genesis of breast cancer. However, some subtypes of alternative BRCA1 splicing are correlated to breast cancer [13]. The occurrence of AS of BRCA1 focuses on two areas, including exons 2, 3, 9 and 10. This phenomenon prompts that the genesis of breast cancer may be promoted by splicing BRCA1. [14]. The mechanisms of generating the breast cancer susceptibility gene BRCA2 mRNA variant may be BRCA2 dysregulated in steroid receptor-negative breast cancer tissues [14].

### Alternative splicing of Rac1

As a kind of GTPase, Rac1 (Ras-related C3 botulinum toxin substrate 1) is a Rho (Ras homologue) family member of downstream effectors. Just like Ras, Rac1 is activated when it is bound with GTP and inactivated when it is bound with GDP [15]. The main mutation of the activated Rac1 may result in neoplastic transformation. Recent research has shown that Rac1 impacts cell proliferation and its expression is higher in breast cancer tissues [16, 17]. Rac1b is produced as a splice variant. Compared with Rac1, Rac1b is more active. [16]. Rac1b may upregulate the expression of cyclin D1 and promote the cell cycle progression [18]. Further research remains to be conducted to explore whether Rac1b promotes breast cancer in the above way or not.

### Alternative splicing of KLF6

Kruppel-like factor 6 (KLF6) is a tumour-suppressing protein [19]. The transcription factor KLF6 gene has been identified as a tumor suppressor because of its inactivation in several types of cancers. Ozdemir F et al have found that KLF6 expression was reduced in a majority of breast cancer patients [20]. Except for full-length KLF6, the AS of other types of KLF6 can produce a dominant negative splice variant known as KLF6-SV1 to inhibit their effects as tumor suppressors. [21, 22]. Hatami R et al. report implicated that KLF6-SV1, as a key driver of breast cancer metastasis, distinguishes with indolent and lethal early-stage cancer and provides a potential therapeutic target for invasive breast cancer [23]. It has been discovered that the high expression of KLF6-SV1 is correlated to more metastatic potential of breast cancer and lower survival rate [23]. It is clear that KLF6-SV1 induces invasion and metastasis of breast cancer. Therefore, it may be used as a potential target for treating invasive breast cancer.

### Alternative splicing of CD44

CD44 (cluster of differentiation 44) is a cellular protein that has been studied in relation to carcinogenesis over the last decade. Tumors of epithelial origin express CD44 in multiple isoforms called variants [24]. In addition to some conflicting data implicated that CD44 have the opposite effects (tumor suppression and tumor promotion). CD44 has been detected to promote protumorigenic expression and promote metastasis. On the other hand, CD44 has been shown to suppress the growth and metastasis of breast cancer [25]. CD44 gets involved in some cellular processes, including lymphocyte homing, adhesion, migration and cell growth regulation, its involvement in these processes is possibly related to its AS [26–28].

The alternative exons may combine in different ways to form more than 20 kind of CD44 variants, while their expressions are detected in various tumors and associated with the ER status [28, 29]. For example, the expression of the variant CD44v2-v10 is positively correlated to ER or PR status. Meanwhile, the variant CD44v3-v10 has positive correlations with the ER or PR status. Although both CD44 splice isoforms (CD44s and CD44v) play essential roles in breast cancer development, CD44v is more associated with favorable prognosis, such as luminal A subtype, while CD44s is related to poor prognosis, such as HER2 or basal cell subtypes [1]. The aforementioned information about AS which promotes the progression of breast cancer may stimulate proliferation of breast cancer cells and impact drug-resistance of breast cancer cells. It is helpful for choosing suitable intervention targets by exploring representative genes related to AS.

## ALTERNATIVE SPLICING THAT SUPPRESSES BREAST CANCER

### Alternative splicing of ERα

It has been found that the expression of ERα46 is downregulated in TAM-resistant breast cancer cells, and the overexpressed ERα46 in MCF-7 cell (a kind of breast cancer cell line) may cause downregulated expressions of several E_2_-induced genes that can stimulate cell proliferation [30]. Thus, it is clear that ERα46 may prevent breast cancer. It may be discovered that ERα46 and ERα36, as two transcripts of AS, are effective for suppressing and promoting breast cancer respectively. It is possible to predict if breast cancer is resistant to TAM based on expression levels of both ERα46 and ERα36.

### Alternative splicing of ERβ

Compared with ERα, ERβ is a gene for coding another category of ER. The ectopically expressed ERβ may inhibit the proliferation of breast cancer cells [31], reduce mobility and invasion of breast cancer cells [32], and suppress tumor formation abilities of MCF-7 in mice [33], therefore ERβ may counteract the effects of ERα for promoting tumor development. ERβ has two variants of AS, including ERβ1 and ERβ2, with different expressions on normal epithelial and non-epithelial parts of breast cancer cells and tissues, which indicates that they play different biological roles in normal tissues and transformed cells [34, 35]. The research of Honma et al has suggested that the expression of ERβ1 is positively correlated to better survival of ERα/PR negative patients with breast cancer and those with triple negative breast cancer who have received adjuvant therapies of TAM [36]. It also has been found in related research that ERβ1 may target IRE1/XBP-1 pathway to promote the apoptosis of breast cancer cells [37]. On the contrary, the disease-free survival (DFS) and overall survival (OS) are poor in ERβ2 positive patients with breast cancer [38]. Above all, ERβ1 and ERβ2 have different impacts upon the survival of breast cancer. In particular, ERβ1 is highly effective for suppressing breast cancer.

### Alternative splicing of VEGF-A and FGFR1

VEGF-A (vascular endothelial growth factor-A) is well known for its key roles in blood vessel growth, has many alternative splice variants, including VEGF121b and VEGF165b, which are less effective for promoting angiogenesis than ordinary VEGF-A [39]. VEGF is a kind of promotors for tumor angiogenesis, and is elevated in breast cancers [40]. VEGF-A can promote a wide range of functions, including adhesion, survival, migration and invasion [41]. The expressions of VEGF121b and VEGF165b are much higher in breast cancer tissues than those in normal mammary tissues, it is necessary to further demonstrate if these two variants can be used as targets for treating breast cancer.

Fibroblast growth factors (FGFs) and their receptors (FGFRs) can regulate numerous cellular processes. Deregulation of FGFR signalling is observed in a subset of many cancers, making activated FGFRs a highly promising potential therapeutic target [42]. FGFR aberrations and gene amplifications lead to increased FGFR signaling and have been linked with poor prognosis and resistance to breast cancer treatments. Aberrant FGFR pathway amplification may promote some breast cancers. Inhibition of FGFR signaling is being explored in the clinic, and data from these trials may help us to select patients who would best respond to these treatments [43]. After AS of FGFR1, FGFR1-α and FGFR1-β would be produced as two variants [44]. FGFR1-β may promote the metastasis of breast cancer cells in mice, however, the metastasis of breast cancer cells is suppressed by FGFR1-α, so it is clear that FGFR1-α is effective for inhibiting breast cancer by resisting FGFR1-β [45]. In a word, it will be helpful for intervening with breast cancer by above AS channels if these ways can be further explored.

## RELATIONSHIPS BETWEEN SPLICEOSOMAL PROTEINS AND BREAST CANCER

As RNA-binding proteins, splicing factors interact with specific RNA sequences. Once they are bound with pre-mRNA, they may guide or block interactions between spliceosomes and pre-mRNA [46]. Some splicing factors (spliceosomal proteins) may activate or inhibit AS according to the sequences binding with them. Up till now, more than 30 kinds of spliceosomal proteins have been discovered in human cells and tissues [47, 48]. These factors are divided into two families, including serine/arginine-rich proteins (SRs) and heterogeneous nuclear ribonucleoproteins (hnRNPs). SRs tend to define exons or introns by splicing enhancer sequences, and recruit spliceosomes to bind with pre-mRNA, in order to promote AS [49]. Conversely, hnRNPs splice silencer sequences with exons and/or introns can inhibit the splicing process. At present, several kinds of SRs have been discovered, among which SRSF1, 2, 3, 5 and 6 are overexpressed in breast cancer [50, 51].

These proteins may regulate the RNA splicing processes of multiple kinds of genes to impact cell cycle regulation, cell proliferation, apoptosis, epithelial-mesenchymal transition, angiogenesis and drug resistance [52, 53]. Belonging to another family of splicing factors, hnRNPs also get involved in regulating mRNA transport, stability and translation [54]. Research has suggested that hnRNPs mainly inhibit AS by several mechanisms as follows: (1) They compete with SRs for binding sites to block spliceosomes and bind with pre-mRNA. (2) They interact with each other to change the structure of pre-mRNA, in order that spliceosomes can't be exposed in certain RNA regions [49]. hnRNPs have been reported to not only have some effects on tumor progression, including inhibiting apoptosis, promoting EMT, metastasis and angiogenesis, but also affect AS to control these important processes [55]. Several kinds of hnRNPs such as hnRNP A1, A2, I and K are overexpressed in breast cancer, which indicates that it is helpful for preventing the emergence of variants related to breast cancer by intervening with expressions or functions of splicing factors.

## RELATIONSHIPS BETWEEN OTHER RNA-BINDING PROTEINS AND BREAST CANCER

Furthermore, some other RNA-binding proteins get involved in the AS directly or indirectly to impact breast cancer. Although Sam68 is overexpressed in breast cancer cells and tissues, p27 and p21 (cell cycle inhibitors) levels will increase if the expression level of Sam68 is too low [56]. p27 was discovered as an inhibitor of cyclin E-CDK2 (E-cyclin-dependent kinase 2), but has been shown to play dual roles to both promote and inhibit cell cycle progression [57]. P21 is a unique marker and the major mediator through which P53 gives the growth arrest command. In addition, the P21 gene singly can lead to apoptosis or cell death [58]. Src-associated substrate during mitosis of 68 kDa (Sam68) is an RNA-binding protein that was the first identified substrate for Brk phosphorylation *in vivo*. Sam68 belongs to the heteronuclear ribonucleoprotein particle K (hnRNP K) homology (KH) domain family of RNA-binding proteins. Sam68 is also a member of the signal transduction and activation of RNA (STAR) family of proteins [59]. Sam68 may induce the formation of D1b, a splice variant cyclin, which promotes tumor progression [60]. Once Sam68 is activated by GTPase RAS, some invariants (including CD44 of Exon V5) will be produced to promote tumor progression [61]. Overexpressed in breast cancer, Y-box binding protein 1 (YB-1) can be specifically bound to the A/C-rich pre-mRNA regions of CD44 to stimulate the expression of the alternative Exon V4 [61].

Fox2 (Forkhead box protein 2) is overexpressed in breast cancer cells [62] and one of its target genes is FGFR2. The conversion between FGFR2 (IIIb) and FGFR2 (IIIc) are closely connected with EMT and MET (mesenchymal-epithelial transition) of breast cancer cells. It has been found that Fox2 (also known as RBM9) upregulates the expression of the Exon IIIb in FGFR2 [63]. The plot of the expression levels of Fox2 in Lapuk A et al. research shows that Fox2 expression is significantly elevated in basal and claudin-low subtypes compared to luminal subtype cells. This observation suggests that Fox2 is an important regulator of subtype specific splicing differences between luminal and basal/claudin-low subtypes. In addition, Fox2 may produce composites with hnRNP H and hnRNP F to inhibit the formation of FGFR2 (IIIc) [64]. The expression of RBM9/FOX2 is not associated with metastatic relapse in breast cancer. But the expression of hnRNP A1 is associated with metastasis in breast cancer. The hnRNP A1 binds to G-quadruplex (G4) RNA elements in the RON/MTS1R 5′UTR. RON encodes a tyrosine kinase receptor known for its function in tumor dissemination and a correlation exists between protein levels of hnRNP A1 in breast cancers [65]. The splicing model revealed a regulatory RNA map for FOX2 to activate or repress AS when bound downstream or upstream of the alternative exon, respectively. The AS of the FOX2 pre-mRNA may result in unique target pre-mRNA splicing regulation. The splicing regulator FOX2 may regulate AS of SRs, hnRNPs and itself [66]. Further research is needed for confirming if these Fox2-regulated splicing processes have effects on genesis and development of breast cancer or not when Fox2 is overexpressed.

EMT plays a critical role during malignant transformation. EMT has been shown to be associated with tumor progression and metastasis. During this process in breast cancer, a crucial role is played by AS. The most EMT-associated AS events are regulated by one or more members of the RBFOX, MBNL, CELF, hnRNP, or ESRP classes of splicing factors. Expression of EMT-associated alternative mRNA transcripts was also observed in primary breast cancer samples, indicating that EMT-dependent splicing changes occur commonly in breast cancer. Shapiro IM et al suggest that splicing regulation can drive critical aspects of EMT-associated phenotypic changes. The molecular description may aid in the development of new diagnostic and prognostic markers for analysis of breast cancer progression [67]. Results of Fici P et al's study showed that the ratio between ESRP1 or ESRP2 and RBFOX2 significantly decreased during EMT and positively correlated with the EMT-specific phenotype in breast cell models, representing promising prognostic markers. Low ESRP1/RBFOX2 ratio value was associated with a higher risk of metastasis in early breast cancer patients [68].

## CONCLUSION

AS has significant effects on genesis and development of breast cancer. Some AS processes may promote breast cancer, whereas some others may suppress breast cancer. Meanwhile, the proteins involved in AS and some others that can bind with mRNA impact breast cancer directly or indirectly. Research has suggested that perhaps the abnormal AS events, extremely active splicing factors or splicing related proteins would be not only developed into markers and targets for diagnosing and intervening with breast cancer, but are also valuable for prognosis of breast cancer.

So many downstream events are impacted by splicing factors that precautions must be taken against intervening with breast cancer, so as to reduce adverse side effects. Comparatively, the abnormal AS in breast cancer, including intervention with ERα and ERβ, would directly suppress breast cancer. Besides, the abnormal splicing possibly differs in breast cancer of different molecular subtypes. It has great value to prevent the emergence of variants related to breast cancer by intervening with expressions or functions of splicing factors. In this paper, we have mentioned some genes may be used as potential targets for treating breast cancer including KLF6-SV1, CD44, VEGF121b, VEGF165b, VEGF-A, FGFR1, hnRNP A1, A2, I and hnRNP K. Among of them VEGF series variants have the more possibility to be used in the clinical practice. But we have no evidence about a certain drug for targeting splicing variant to intervent with the invasion or metastasis of breast cancer patients. But the deep research can help the clinical practice through searching the new drugs and better patient stratification, and then improve the therapeutic effects. Thus, it will be interest to explore how to more effectively diagnose and intervene with breast cancer of various subtypes in the future.
